# Rapamycin enhances survival in a *Drosophila* model of mitochondrial disease

**DOI:** 10.18632/oncotarget.12560

**Published:** 2016-10-11

**Authors:** Adrienne Wang, Jacob Mouser, Jason Pitt, Daniel Promislow, Matt Kaeberlein

**Affiliations:** ^1^ University of Washington, Department of Pathology, Seattle, WA, USA; ^2^ University of Washington, Department of Biology, Seattle, WA, USA

**Keywords:** mitochondria, leigh syndrome, rapamycin, Drosophila, complex I

## Abstract

Pediatric mitochondrial disorders are a devastating category of diseases caused by deficiencies in mitochondrial function. Leigh Syndrome (LS) is the most common of these diseases with symptoms typically appearing within the first year of birth and progressing rapidly until death, usually by 6-7 years of age. Our lab has recently shown that genetic inhibition of the mechanistic target of rapamycin (TOR) rescues the short lifespan of yeast mutants with defective mitochondrial function, and that pharmacological inhibition of TOR by administration of rapamycin significantly rescues the shortened lifespan, neurological symptoms, and neurodegeneration in a mouse model of LS. However, the mechanism by which TOR inhibition exerts these effects, and the extent to which these effects can extend to other models of mitochondrial deficiency, are unknown. Here, we probe the effects of TOR inhibition in a *Drosophila* model of complex I deficiency. Treatment with rapamycin robustly suppresses the lifespan defect in this model of LS, without affecting behavioral phenotypes. Interestingly, this increased lifespan in response to TOR inhibition occurs in an autophagy-independent manner. Further, we identify a fat storage defect in the ND2 mutant flies that is rescued by rapamycin, supporting a model that rapamycin exerts its effects on mitochondrial disease in these animals by altering metabolism.

## INTRODUCTION

Childhood mitochondrial diseases are caused by deficiencies in mitochondrial function that arise from an array of mutations in genes coding for mitochondrial proteins [1, 2]. Patients suffering from mitochondrial disease can exhibit a range of pathologies across tissues, including neurodegeneration, seizures, ataxia, cardiomyopathy, muscle wasting, hearing loss, learning disorders, respiratory problems, sensitivity to anesthesia and lactic acidosis [3-5].

Mitochondria provide our cells with an advanced system for energy production and maintain a specialized environment for several fundamental metabolic processes such as ATP production and fatty acid oxidation. Hundreds of mitochondrial proteins, both nuclear- and mitochondrially- encoded, are involved in energy metabolism. These proteins make up the mitochondrial electron transport chain (ETC) complexes I, II, III, IV, V, and pyruvate dehydrogenase, which are involved in oxidative phosphorylation and generation of ATP. Mutations in over 70 of these genes have been linked to mitochondrial disease, with the most common deficiencies found in complex I and complex IV [6, 7].

Leigh Syndrome (LS), also known as juvenile subacute necrotizing encephalomyelopathy, is the most common childhood mitochondrial disease [6, 8]. LS can be caused by mutations in several different nuclear and mitochondrially encoded proteins, with symptoms typically appearing during the first few years of life and progressing rapidly until death, usually by 6-7 years of age [6, 9]. Mutations in the complex I assembly factor NDUFS4 and the mitochondrially encoded ND2 subunit of complex I are two of the causal mutations implicated in LS in children. Mice lacking NDUFS4 are deficient for Complex I activity and recapitulate many of the symptoms associated with LS, including retarded growth, necrotizing encephalopathy, and dramatically reduced lifespan [10, 11]. NDUFS4 knockout (KO) mice also display pathological hyperactivation of signaling through the target of rapamycin (TOR) pathway in the brain [12]. This aberrant signaling response is associated with accumulation of pyruvate, lactate, and glycolytic intermediates, and a decrease in free amino acids, free fatty acids, nucleotides and products of nucleotide catabolism [12].

We have recently reported that reducing TOR signaling is sufficient to suppress replicative life span deficits in yeast strains with mutations in disease-associated mitochondrial genes [13], and that inhibiting the aberrant activation of TOR by administration of rapamycin significantly rescues the shortened lifespan, neurological symptoms, and neurodegeneration in NDUFS4 KO mice [12, 14]. Rapamycin is an FDA approved drug used clinically to treat transplant patients and to augment anticancer treatments [15, 16]. It inhibits TOR, a serine/threonine kinase that integrates environmental cues from nutrients and growth factors to regulate cell growth [17, 18]. In favorable growth conditions, TOR is active and cells undergo organelle biogenesis, protein synthesis and proliferation. Inhibition of TOR activity through reduced nutrient signaling or treatment with rapamycin decreases translation and induces autophagy, allowing the cell to increase bulk protein and organelle degradation in order to maintain ATP production in the absence of external nutrients [18]. Interestingly, rapamycin does not rescue mitochondrial ETC assembly and function in the NDUFS4 KO mice, but does appear to suppress the accumulation of glycolytic intermediates in the brain, perhaps by shifting metabolism away from glycolysis and towards fatty acid mobilization, ketogenesis, and amino acid catabolism [12]. The mechanism by which TOR inhibition exerts these effects and the extent to which these effects can extend to other models of mitochondrial deficiency are unknown.

A *Drosophila* model of LS has been recently described in which the Complex I subunit ND2 is compromised by a 9-nucleotide deletion [19]. These flies exhibit decreased Complex I assembly and function, heat sensitivity, flight deficits, a mechanically induced paralysis termed “bang-sensitivity”, increased sensitivity to hypercapnea and hypoxia, neurodegeneration, and decreased lifespan. Here, we take advantage of this genetic model system to understand the mechanistic basis of rapamycin's effects and to investigate the interaction between TOR signaling and disease progression in this *Drosophila* model of mitochondrial disease. As in mice, we find evidence for hyperactivation of TOR in Complex I deficient flies. Treatment with rapamycin robustly extends the lifespan deficiency in this model of LS without affecting behavioral phenotypes. Interestingly, this increased lifespan in response to TOR inhibition occurs in an autophagy-independent manner. Further, we observe a fat storage defect in the ND2 mutant flies that is rescued by rapamycin, similar to what we have previously observed in mice, and supporting the model that rapamycin exerts its effects by altering carbon metabolism rather than directly suppressing the defect in mitochondrial Complex I function.

## RESULTS

### Rapamycin extends lifespan in a *Drosophila* model of complex I deficiency

**Figure 1:**
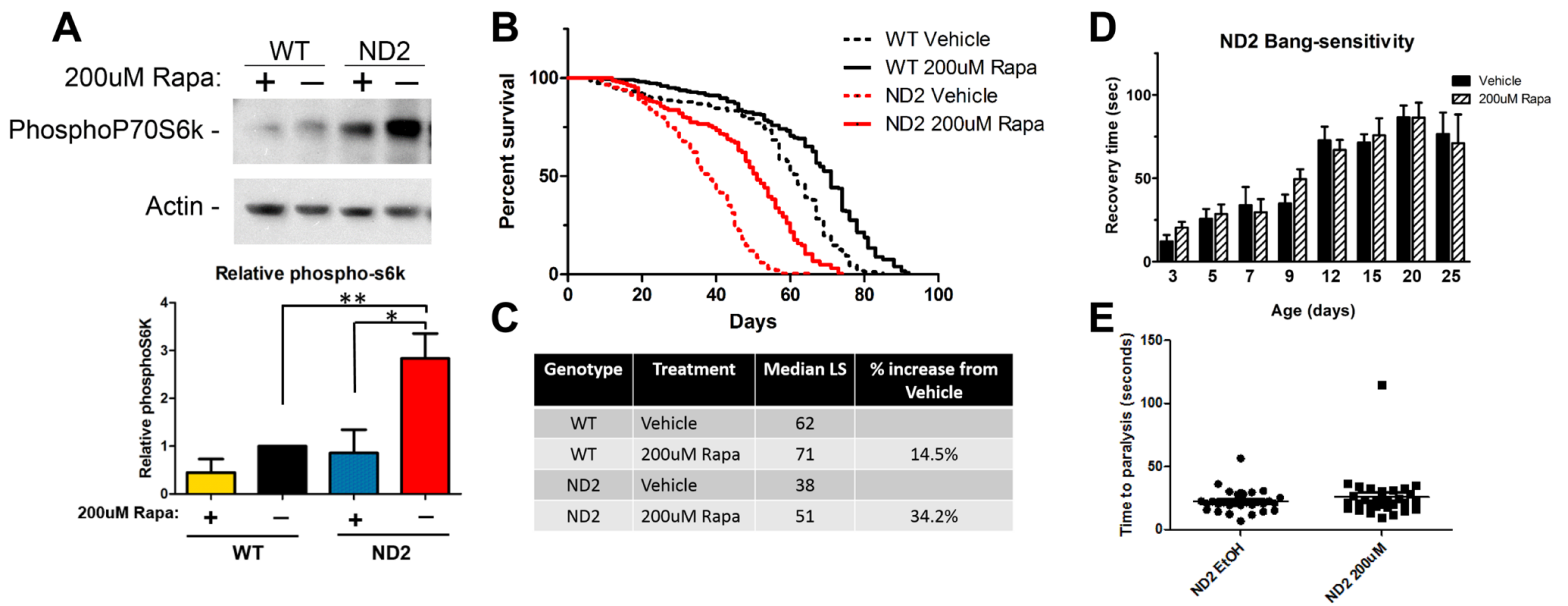
TOR inhibition by rapamycin increases lifespan in ND2 mutant flies **A.** ND2 mutant flies exhibit increased phosphorylation of the TORC1 target p70S6k as compared to WT. Rapamycin abolishes TORC1 signaling. Actin is shown as control and quantification of biological triplicates is shown below. Significance was determined by ANOVA *p < .05 **p < 0.01. **B.** ND2 mutant flies exhibit a significant reduction in lifespan (red dashed line) in comparison to WT flies (black dashed line). Rapamycin treatment robustly extends lifespan in mtND2 flies (red solid line) as well as WT flies (black solid line). **C.** Quantification of median lifespan. ND2 flies have a 38% reduction in median lifespan compared to WT flies, and rapamycin robustly increases median lifespan in mtND2 flies by 34.2%. WT flies treated with rapamycin show a more modest increase in lifespan of 14.5%. **D.** ND2 mutant flies exhibit a mechanical-stress induced paralysis (solid black bars). Time to recovery after mechanical stress worsens with age and is not affected by rapamycin treatment (hashed black bars). WT flies show no paralysis, and only ND2 flies are shown. **E.** 15-day-old ND2 mutant flies exhibit heat-induced paralysis that is unaffected by rapamycin treatment. WT flies did not paralyze in the timeframe of this experiment; only ND2 flies are shown.

To first define the interaction between TOR signaling and mitochondrial disease in the ND2 flies, we tested the effect of TOR inhibition on lifespan in this disease model. Treatment with 200uM rapamycin was sufficient to inhibit phosphorylation of the TOR target p70s6K (Figure 1A), which was elevated in the ND2 flies compared to untreated wild type (WT) animals. Rapamycin treatment robustly extended the lifespan of both WT and ND2 mutant flies (Figure 1B, 1C). However, the percentage of lifespan extension was greater for the ND2 flies (34.2%) than for the WT controls (14.5%), similar to what we previously reported in NDUFS4 KO mice [12].

To determine the extent to which rapamycin rescues other deficits in ND2 flies, we evaluated the effects of rapamycin on behavioral phenotypes exhibited by these animals. ND2 flies exhibit “bang-sensitivity” in which mechanically induced stress results in a short-term paralysis quantified by measuring the length of time it takes for each individual fly to right itself. This phenotype is apparent in the ND2 flies by 15 days of age. While wild type flies do not show bang sensitivity, 15-day-old ND2 mutant flies take an average of 71 seconds to right themselves (Figure 1D). Treatment with rapamycin does not significantly alter the average time it takes to recover from paralysis (Figure 1D). ND2 flies also exhibit heat sensitivity, in which exposure to 37°C leads to paralysis. WT flies do not exhibit this sensitivity to heat. Treatment of ND2 flies with rapamycin has no effect on their susceptibility to heat-induced paralysis (Figure 1E). These results indicate that the effects of rapamycin on these behavioral phenotypes can be dissociated from the life-extending effects of TOR inhibition.

### Rapamycin does not rescue susceptibility to oxygen stress, but does enhance recovery from anoxia

**Figure 2:**
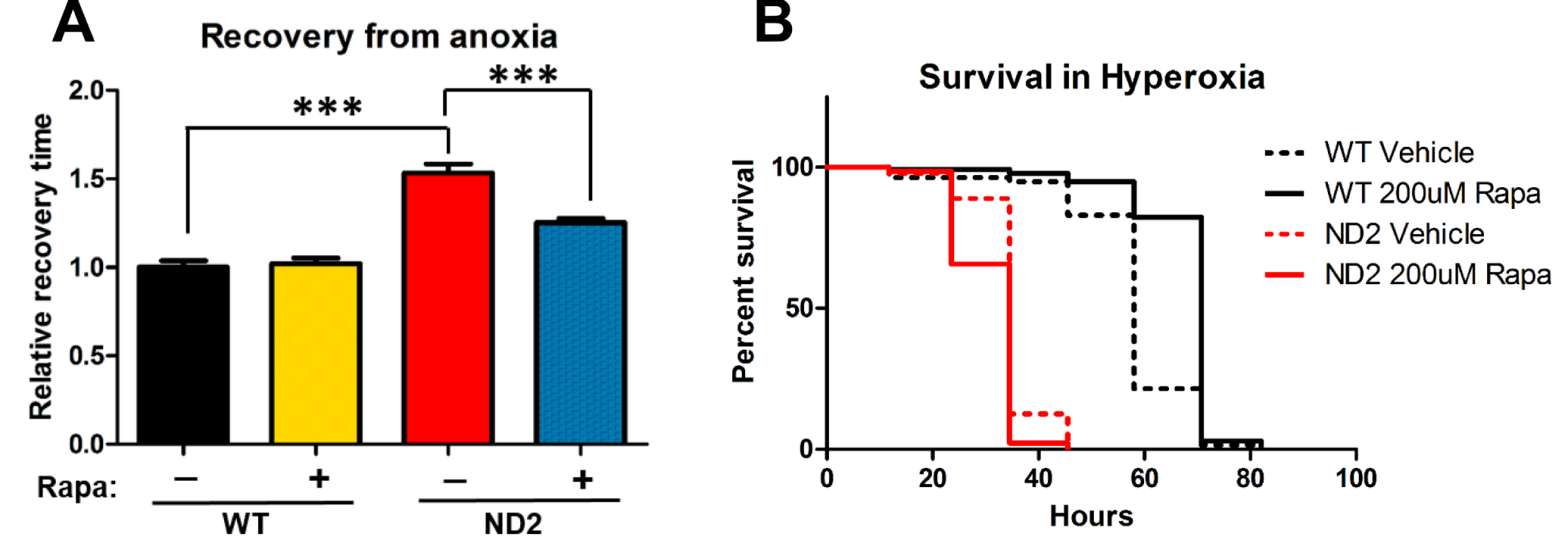
Effects of rapamycin on response to oxygen stress **A.** ND2 mutant flies take longer to recover from anoxia-induced paralysis compared to WT controls. Rapamycin partially rescues this phenotype, but not to WT levels. Data are represented as mean +/- SEM. Significance was determined by ANOVA ***p < 0.001. **B.** Survival in 99.9% O_2_. ND2 mutant flies are more susceptible to hyperoxia (solid red line) and rapamycin has no effect on response to hyperoxic stress (dashed red line).

Because mitochondria are central in the consumption of oxygen and the production of energy, it is unsurprising that changes in atmospheric oxygen have been shown to modify disease phenotypes in NDUFS4 KO mice, and in a worm model of mitochondrial disease [10, 20]. To investigate the extent to which rapamycin rescues sensitivity to extremes in oxygen levels in this *Drosophila* model of complex I inhibition, we measured sensitivity of ND2 mutant flies to anoxia and hyperoxia. Exposing 15-day-old WT and ND2 mutant flies to anoxia for 2 minutes causes both WT and ND2 flies to enter into a hypoxia-induced paralysis. The time it takes for flies to succumb to hypoxia-induced paralysis was unaffected by genotype or treatment (Supplemental Figure S1). ND2 flies, however, exhibit a deficit in recovery from this anoxic insult, taking 53% longer on average to recover once exposed to room air (Figure 2A). ND2 flies are also more susceptible to hyperoxia. When 15-day-old flies are placed in normobaric hyperoxia (101 KPa oxygen), WT flies have a median survival of 58 hours while survival of ND2 flies is reduced to 34.5 hours (Figure 2B), consistent with increased oxygen toxicity due to decreased ETC function.

Rapamycin treatment had no effect on the time it took for ND2 mutant flies to succumb to anoxia-induced paralysis, nor on the length of survival in hyperoxia (Figure 2A, Figure 2B), but interestingly, did enhance the recovery of ND2 flies from anoxic insult, with rapamycin treated flies waking 28% faster (Figure 2A).

### Rapamycin extends lifespan in complex I mutants in an autophagy independent manner

**Figure 3:**
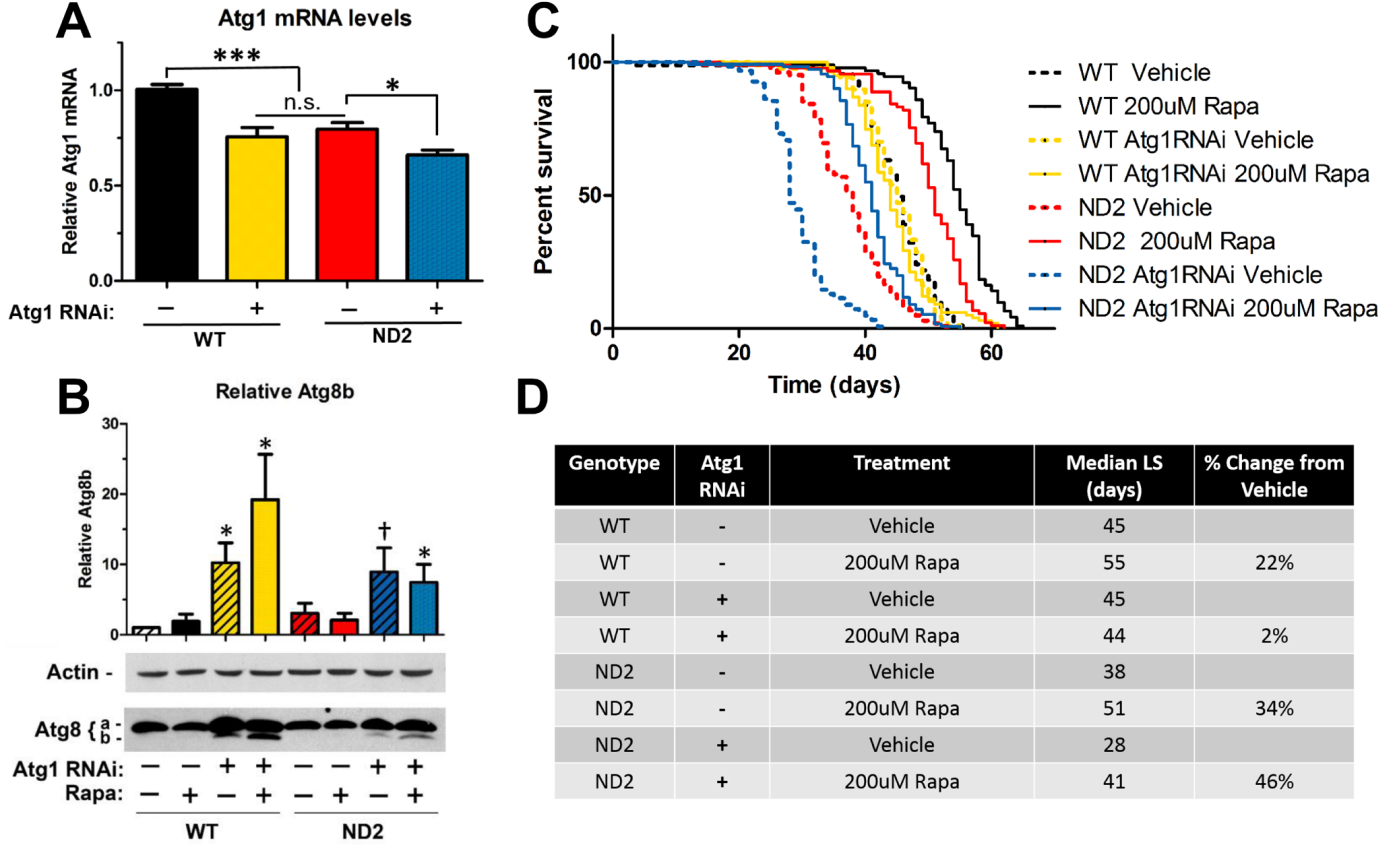
Rapamycin extends lifespan in ND2 mutant flies in an autophagy-independent manner **A.** QPCR on Atg1 mRNA levels. ND2 mutant flies that are heterozygous for the Atg1RNAi transgene but not the Gal4 driver show decreased levels of Atg1 mRNA as compared to WT. Expression of Atg1 RNAi driven by Hsp70-Gal4 driver results in a further decrease in Atg1 mRNA levels. Data are represented as mean value normalized to geometric mean of three housekeeping genes +/- SEM. **B.** Western blot analysis of the autophagic marker Atg8. Upper band is Atg8a (non-lipidated form) and lower band is Atg8b (lipidated form). Accumulation of the lipidated form (Atg8b) as seen in flies expressing Atg1RNAi and the Hsp70Gal4 driver (Lanes 3,4,7 and 8) is associated with a blockage in autophagy [39]. *p < 0.05, † 0.05 < *p* < 0.1. **C.** Lifespan analysis of WT and ND2 flies that are heterozygous for the Atg1 RNAi transgene alone (WT: black, ND2: red), or heterozygous for the Atg1 RNAi transgene and the Hsp70-Gal4 driver (WT: Green, ND2: Blue) on vehicle supplemented control food (dashed lines) or food supplemented with 200uM rapamycin (solid lines). Knockdown of autophagy in WT flies (yellow dashed line) abolishes the lifespan extension by rapamycin in WT flies (yellow solid line, *p* > .0.05). ND2 flies expressing Atg1 RNAi (dashed blue line) exhibit a significant decrease in lifespan compared to ND2 flies (dashed red line, ***:*p* < .001), but still prove responsive to rapamycin (solid blue line, ***:*p* < 0.001). **D.** Quantification of median lifespan.

One well-characterized effect of TOR inhibition is the induction of autophagy, a mechanism of bulk degradation in which cytoplasmic cargo are engulfed by double-membraned autophagosomes and trafficked to the lysosome for degradation. In nutrient-rich conditions, TOR phosphorylates and inhibits Atg1, a key kinase that initiates the formation of autophagosomes [21]. Inhibition of TOR releases its inhibition on Atg1 and increases autophagic flux. Experiments in flies have shown that Atg1 also has inhibitory effects on TOR, creating a negative-feedback loop [22, 23]. In agreement with our data indicating hyperactive TOR signaling in our ND2 model, ND2 mutant flies also exhibit decreased Atg1 expression (Figure 3A). To test the extent to which rapamycin is extending lifespan by increasing autophagy, we took advantage of the *Drosophila* Gal-4/UAS system [24] to knock down autophagy in wild type and ND2 mutant flies by expressing dsRNAi to Atg1. Expression of Atg1 RNAi by the ubiquitous actin driver resulted in embryonic lethality (data not shown). However, expressing Atg1 RNAi under the Hsp70 Gal4 driver at 25C allowed flies to overcome developmental autophagy requirements and reach adulthood, at which point the flies were reared at 29C for the remainder of their lifespan. QPCR analysis confirmed a modest decrease in Atg1 mRNA levels in those flies expressing the Atg1 RNAi (Figure 3A), however even this modest decrease was enough to result in a significant defect in autophagy as seen by accumulation of LC3II (Figure 3B).

While knockdown of autophagy in control flies did not affect lifespan, a similar degree of autophagy inhibition significantly decreased lifespan in ND2 flies (Figure 3C), indicating that activation of autophagy is critical to survival in this model of mitochondrial disease. As has been previously reported, lifespan extension from rapamycin in control flies is autophagy dependent [25], and our results support this conclusion. Interestingly, lifespan extension by rapamycin was not abolished by decreased autophagy in the ND2 mutant flies. This supports a model in which rapamycin is targeting some deficit in ND2 mutant flies to extend lifespan independent of autophagy induction.

### ND2 flies exhibit a fat storage defect that is rescued by rapamycin

**Figure 4:**
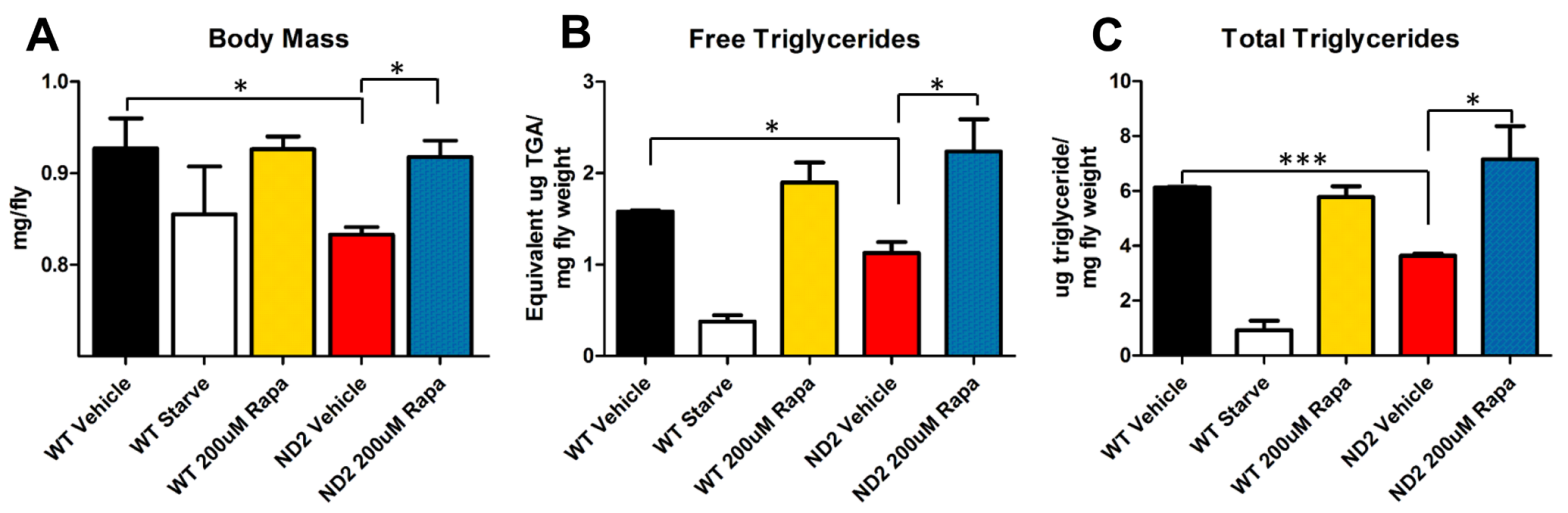
ND2 mutant flies exhibit defects in fat metabolism that are reversed by rapamycin **A.** Average weight per fly. 25-day-old ND2 flies exhibit a decrease in body mass that is rescued to WT levels by treatment with rapamycin. **B.** Free triglyceride levels. ND2 flies show decreased levels of free triglycerides that are rescued by treatment with rapamycin. **C.** Total triglyceride levels per mg of fly weight. ND2 mutant flies also exhibit a decrease in total triglyceride levels that are significantly increased by rapamycin treatment. Data are represented as mean +/- SEM. Significance was determined by ANOVA *p < .05, **p < 0.01, ***p < 0.001.

Humans affected with Leigh syndrome exhibit significant metabolic defects, including accumulation of lactate, pyruvate, and intermediate metabolites consistent with problems in amino acid catabolism [4, 26]. In a mouse model of Leigh syndrome, there is evidence that rapamycin changes metabolic processes, shifting metabolism away from glycolysis [12]. TOR serves as a central regulator of energy metabolism and rapamycin has been shown to increase beta-oxidation and increase catabolism of free fatty acids [27]. Indeed, ND2 flies show a significant reduction in body mass in comparison to control flies, and ND2 flies reared on rapamycin-supplemented food show a rescue to almost wild type weight (Figure 4A). Because of TOR's role in regulating metabolism, we measured triglyceride levels in ND2 and WT flies treated with vehicle or rapamycin. ND2 flies exhibit a decreased level of free triglycerides, indicating a deficiency in the ability to mobilize and metabolize fat (Figure 4B). Additionally, ND2 flies also exhibit decreased total triglyceride levels in comparison to controls (Figure 4C), indicative of a significant defect in fat storage. These phenotypes are suppressed by treatment with rapamycin, similar to what we have observed in the NDUFS4 KO mice.

## DISCUSSION

TOR signaling lies at the nexus of nutrient sensing and metabolism, integrating numerous environmental signals into cellular responses that regulate autophagy, translation, mitochondrial metabolism, and entry into the cell cycle [17, 18]. Inhibition of TOR signaling by rapamycin has been shown to extend lifespan in wild type populations of numerous species [16, 28], and in mouse and worm models of mitochondrial disease [12, 14, 29]. Results from our group and others support a role for rapamycin in metabolic reprogramming, rescuing energy deficits in mitochondrial disease. Here, we demonstrate that a treatment that ameliorates disease symptoms and increases lifespan in a mouse model of mitochondrial disease is also effective in a genetically distinct model. We show genotype-specific effects of rapamycin in mitochondrial disease and evidence of metabolic changes that may underlie the lifespan extension.

Patients with mitochondrial disease exhibit metabolic defects such as lactic acidosis and exercise intolerance [30, 31]. Similarly, NDUFS4 KO mice exhibit a defect in fat storage, accumulation of glycolytic intermediates, and elevated lactic acid levels, all of which are reversed by treatment with rapamycin [12]. This is consistent with dysregulated TOR signaling, as TOR has been shown to regulate adipogenesis [32] and can activate more than thirty genes that regulate the synthesis and uptake of fatty acids, sterols, triglycerides, and phospholipids through its activation of sterol regulatory element-binding protein (SREBP) [33, 34].

Our data support a model in which metabolic deficiencies in mitochondrial disease caused by hyperactive TOR signaling lead to increased mortality and suggest that rapamycin exerts its lifespan-extending effects by altering metabolism. ND2 flies have reduced body weight and defects in fat storage and metabolism, all symptoms that are reversed by rapamycin. Additionally, knockdown of autophagy in the ND2 background further reduces lifespan, an effect not seen in our WT flies under identical conditions. This implies that ND2 flies rely more upon basal autophagy than WT flies, perhaps as an alternative energy source, and is consistent with our hypothesis that lifespan in ND2 flies is decreased due to metabolic deficits.

Notably, our results also indicate that enhanced autophagy is not required for the lifespan extending effects of rapamycin in this fly model of mitochondrial disease. Inhibition of autophagy in WT flies abolishes the lifespan extending effects of rapamycin [25]. However, ND2 flies deficient in autophagy still respond to rapamycin treatment with a robust lifespan extension (Figure 3).

Although we favor a metabolic explanation, an alternative possibility is that rapamycin rescues the survival defect of ND2 flies by reducing global mRNA translation. TOR activity promotes translation, and studies in *C. elegans* have indicated that translation inhibition can suppress some mitochondrial defects in a manner similar to rapamycin treatment [29]. One argument against this possibility is the reduced weight of the ND2 flies despite hyperactive TOR signaling. This reduction in size is reversed by rapamycin treatment. However, additional studies will be required to definitively assess whether effects of rapamycin on mRNA translation are involved in the observed suppression of mitochondrial disease in the ND2 flies.

It is particularly interesting that many, but not all, phenotypes associated with the ND2 mutation are suppressed by rapamycin. Fat storage, recovery from anoxia, and autophagy-independent survival all show significant improvements from rapamycin treatment specific to the ND2 genotype, and indicate fundamental deficiencies in mitochondrial disease that are rescued with rapamycin. In contrast, bang sensitivity, heat sensitivity, survival in hyperoxia and susceptibility to anoxia-induced paralysis are ND2 phenotypes that were not impacted by rapamycin. While mitochondrial disease is rare, mitochondrial dysfunction is thought to underlie many more common neurological conditions such as Parkinson's disease and Alzheimer's disease. The extent to which rapamycin's effects on metabolism may affect disease progression in these more common neurodegenerative disorders will be of interest for future study.

Our results further support the potential of TOR inhibition as a therapeutic strategy in diseases arising from mitochondrial dysfunction [35]. However, TOR signaling affects a myriad of cellular processes, and identification of the specific mechanisms by which hyperactive TOR signaling contributes to disease pathogenesis and the mechanisms by which rapamycin treatment rescues lifespan in mitochondrial disease is needed. Our data identify a potential role of TOR inhibition as a powerful tool in combinatorial therapies for this as-of-yet untreatable family of diseases. Further, our work demonstrates the potential that *Drosophila* holds as a useful genetic model system to understand both the molecular and genetic factors influencing mitochondrial disease, as well as the mechanistic underpinnings of successful mitochondrial disease therapies like rapamycin.

## MATERIALS AND METHODS

### *Drosophila* culture and rapamycin treatment

All experimental flies were maintained on cornmeal/molasses medium (0.9% Agar, 7.2% cornmeal, 10% molasses, 2.5% yeast, 3% tegosept, 0.3% propionic acid) at 25° C, except where indicated with a 12-hour light-dark cycle. Experimental food was cooked in batches and stored in the dark at 4C for no longer than two weeks. Rapamycin (LC Laboratories) stock was made fresh each time, at a concentration of 50mM in 100% EtOH and added to food to a final concentration of 200uM after it had cooled to less than 60C. Equal volumes of EtOH alone was added to vehicle control food. Isogenic w1118, ND2 del, and Hsp70-Gal4, and Atg1 RNAi flies were a kind gift from the laboratory of Dr. Leo Pallanck (University of Washington).

### Determination of body weight

Female flies were collected under light CO_2_ and placed directly onto food containing either 200uM of rapamycin or vehicle control at a density of less than 30 flies per vial. Flies were transferred to fresh food every 2 days, and aged to 21 or 25 days before assessing weight. Starved flies were transferred to vials with moist whatman paper for 48 hours prior to collection. Flies in each vial were weighed in groups, and average weight per fly was determined within the group. Each condition was performed in triplicate and each experiment was performed at least three times. Flies were then snap frozen for triglyceride analysis. One-way ANOVA followed by Newman-Keuls multiple comparison test was performed using Graphpad Prism (Graphpad software, LaJolla, CA) to determine statistical significance (*: *p* < .05; **: *p* < 0.01; ***: *p* < 0.001).

### Lifespan analysis

Flies were collected within 48 hours of eclosion and mated for 48-72 hours before being sexed and females were divided into vials containing food supplemented with 200uM Rapamycin or vehicle control. Flies were transferred to new food within 48-72hrs and scored for deaths. Rapamycin food (as above) was prepared fresh every two weeks. For each experiment, at least four vials per condition were used with no more than 30 flies per vial. Developmental density was controlled for by parental crosses with five females per vial for no more than five days. To test for differences between genotypes or treatments, we used the survdiff function in the survival package in R [36]. Lifespans were repeated at least twice, except in the case of the Atg1 RNAi lifespan, in which the ability of rapamycin to enhance survival in the ND2 genotype when autophagy has been inhibited was independently verified (Supplemental Figure S2).

### Atg1 RNAi

ND2 mutant and WT control female flies were crossed to male flies carrying a UAS responsive TRiP transgene to express dsRNAi to Atg1 (Bloomington, #26731) and maintained over the TM6 balancer. Experimental flies were produced by crossing female UAS_Atg1RNAi flies carrying either WT or ND2 mutant mitochondrial DNA to Hsp70-Gal4 carrying males. Sibling flies heterozygous for the UAS transgene only were used as control.

### QPCR analysis

15-day-old flies were snap frozen in liquid nitrogen and RNA was extracted as previously described [37]. cDNA was synthesized using iScript (BioRad Laboratories, Hercules, CA) and QPCR was performed using Taqman primers to Atg1 (ThermoFisher, Dm01802843_m1), RPL32, γ-Tubulin (ThermoFisher, Dm01841764_g1) and Actin 5C (ThermoFisher, Dm02361909_s1). Atg1 levels were normalized to the geometric mean of the three housekeeping genes RPL32, γ-Tubulin, and Actin 5C. One-way ANOVA was performed using Graphpad Prism (Graphpad software, LaJolla, CA.) to determine statistical significance (*: *p* < .05; **: *p* < 0.01; ***: *p* < 0.001).

### Western blot

15-day-old flies were snap frozen in liquid nitrogen and homogenized with a pestle directly into 2x laemmli sample buffer. Homogenates were boiled for 5 minutes at 95 °C and centrifuged briefly at 15,000 rcf. Supernatants were then run on 10%, 12%, or 4%-12% Bis-Tris gels (Nupage) and transferred to nitrocellulose. Blots were labeled with monoclonal antibodies to Actin (Millipore, #CP01), and polyclonal antibodies to phospho-*Drosophila* p70 S6 Kinase (Cell Signaling, #9209) and Atg8 (Millipore, #ABC974). The HRP-conjugated secondary antibodies used were from Santa Cruz Biotechnologies (#SC-2004, SC-2005), and blots were developed using the ECL detection system (Amersham) and the Lumigen ECL Ultra system (Lumigen). Experiments were repeated at least three times, and films were scanned and subjected to densitometric analysis using NIH Image-J. Signal from each experiment was internally normalized to the wild type vehicle control. Data was log-transformed and significance was determined by ANOVA (†: 0.05 < *p* < 0.1; *: *p* < .05; **: *p* < 0.01; ***: *p* < 0.001).

### Heat induced paralysis

15-day-old flies were assayed for heat-induced paralysis by placing groups of ten animals into pre-warmed vials (Genesee) maintained in a 39°C water bath. The time for the flies to become completely paralyzed was recorded. Experiment was run in triplicate (*P* > 0.05).

### Mechanical stress induced paralysis

Flies were assayed for bang sensitivity using a modification of a previously published protocol [38]. Briefly, groups of 10-15 flies were vortexed at max speed for ten seconds in inverted glass vials containing cotton stoppers, and the time required for each individual animal to right itself was recorded.

### Sensitivity to anoxia

Fifteen 15-day-old flies were placed into 100mL Grenier tissue culture flasks fitted with rubber septa (Sigma-Aldrich, St. Louis, MO) and 18-gauge syringe needle gas ports (BD, Franklin Lakes, NJ) without gassing and allowed to acclimate for 15 minutes. 99.995% nitrogen (Praxair, Seattle, WA) was then flowed into the chamber for two minutes at a rate of 400 mL/min using a rotameter (Omega Engineering INC, Stamford, CT), at which point the gas source was switched to room air maintained at the same flow rate. Time for flies to recover from anoxia-induced paralysis once room air began flowing was recorded. Response times were normalized to the same day wild-type control value to account for day-to-day variation in response across both groups.

### Hyperoxia lifespan

Flies were collected and placed on vehicle or rapamycin supplemented food as described above. Vials were maintained in airtight chambers (2.5 L jar, Mitsubishi, Japan) fitted with gas ports (Cole-Parmer, Vernon Hills, IL) at a flow rate of 1L/min delivered *via* rotameter (Omega Engineering INC, Stamford, CT) place in a 25C incubator. Gas was humidified *via* a deionized water-filled gas wash bottle (Bel-Art Products, Wayne, NJ) placed in-line between the rotameter and the fly chamber. Constant flow of either room air or 99.9% O2 (Praxair, Seattle, WA) was maintained throughout the experiment and flies were removed from the chambers for less than 5 minutes and scored for viability every 12 hours.

### Determination of triglyceride levels

Flies were reared as above. Five frozen flies were homogenized in PBS-T buffer. Total triglyceride levels were measured using Infinity reagent and free triglyceride levels were determined using the Serum Triglyceride Determination kit from Sigma (St. Louis, MO). One-way ANOVA followed by Newman-Keuls multiple comparison test was performed using Graphpad Prism (Graphpad software inc.) to determine statistical significance (*: *p* < .05; **: *p* < 0.01; ***: *p* < 0.001).

## Supplemental Material


