# Supplementary material for: Rapamycin enhances survival in a Drosophila model of mitochondrial disease
Source: Oncotarget. 2016 Oct 11;7(49):80131–9. doi: 10.18632/oncotarget.12560 (PMC5348310; doi:10.18632/oncotarget.12560)
Supplement: Supplementary file 1 [file oncotarget-07-80131-s001.pdf]

Rapamycin enhances survival in a *Drosophila* model of mitochondrial disease

Supplemental Material

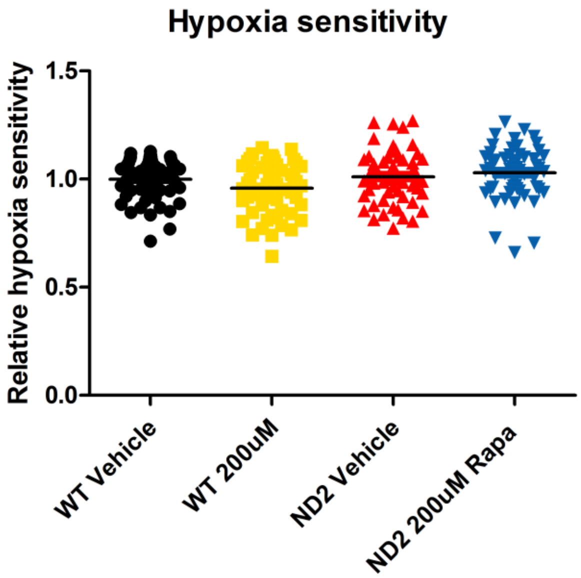

Figure S1. Sensitivity to anoxia-induced paralysis is not affected by genotype or rapamycin treatment. Exposure of 15d old flies to 99.995% nitrogen causes a hypoxia-induced paralysis. Cohorts of fifteen 15 day old flies were placed in airtight flask, allowed to acclimate and then exposed to 99.995% nitrogen. Time to paralysis for each fly was measured and normalized to the mean time of WT Vehicle control to account for day-to-day differences. Experiment was replicated four times.

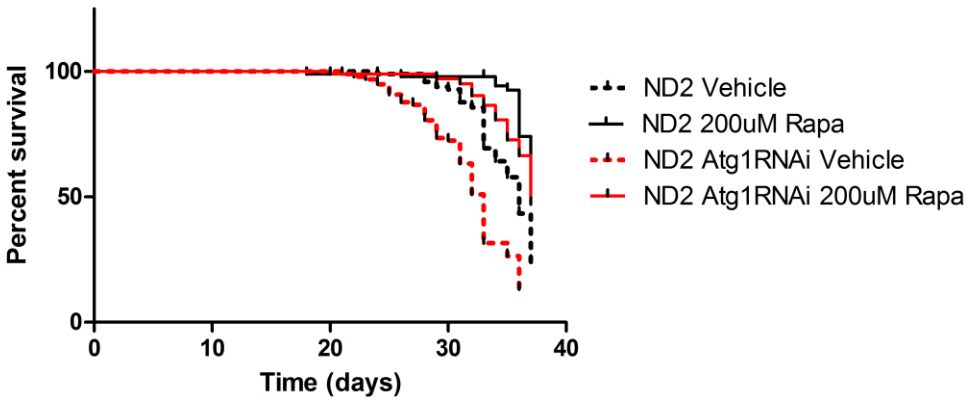

Figure S2. Rapamycin extends lifespan in ND2 mutant flies in an autophagy-independent manner. Independent lifespan analysis supports a decreased lifespan in ND2 mutant flies that have knocked down autophagy (dashed red line) versus ND2 mutant flies heterozygous for only the transgene (dashed black line), $p<0.001$. ND2 flies with knocked down autophagy (dashed red line) are still responsive to rapamycin-induced lifespan extension (solid red line), $p<0.001$. Experiment was truncated due to media contamination. All analyses were carried out prior to contamination and all flies still alive were treated as censored.
